# Audiovisual messages may improve the processing of traffic information and driver attention during partially automated driving: An EEG study

**DOI:** 10.1186/s41235-024-00580-8

**Published:** 2024-09-11

**Authors:** Marina Pi-Ruano, Alexandra Fort, Pilar Tejero, Christophe Jallais, Javier Roca

**Affiliations:** 1 ERI-Lectura (UVEG), Avenida Blasco Ibáñez, 21, 46010 Valencia, Spain; 2 Departamento de Psicología Evolutiva y de la Educación (UVEG), Avenida Blasco Ibáñez, 21, 46010 Valencia, Spain; 3 LESCOT (Univ. Eiffel), 25 Avenue François Mitterrand, Case24. Cité Des Mobilités, 69675 Bron Cedex, France; 4 Departamento de Psicología Básica (UVEG), Avenida Blasco Ibáñez, 21, 46010 Valencia, Spain

**Keywords:** Audio messages, Partial automation, Vigilance, EEG, Variable message signs

## Abstract

Partially autonomous vehicles can help minimize human errors. However, being free from some driving subtasks can result in a low vigilance state, which can affect the driver’s attention towards the road. The present study first tested whether drivers of partially autonomous vehicles would benefit from the addition of auditory versions of the messages presented in variable message signs (VMS), particularly, when they find themselves in a monotonous driving situation. A second aim was to test whether the addition of auditory messages would also produce an indirect effect on the driver’s vigilance, improving performance on other driving subtasks not related to the message processing. Forty-three volunteers participated in a driving simulator study. They completed two tasks: (a) a VMS task, where they had to regain manual control of the car if the VMS message was critical, and (b) a car-following task, where they had to pay attention to the preceding car to respond to occasional brake events. Behavioral and EEG data were registered. Overall, results indicated that the addition of audio messages helped drivers process VMS information more effectively and maintain a higher level of vigilance throughout the driving time. These findings would provide useful information for the development of partially automated vehicles, as their design must guarantee that the driver remains attentive enough to assume control when necessary.

## Significance Statement

During periods of reduced stimulation, such as in low-demand traffic situations or while driving through familiar environments, drivers may be more susceptible to disengagement from the driving task, which would hinder critical information processing and endanger safety on the road. Our research contributes to the current scientific understanding by assessing the benefits of combining auditory messages with variable message sign content in partially autonomous driving contexts. Auditory messages can serve as warning signals, providing an additional layer of alertness that helps drivers maintain a higher level of attention, as well as conveying critical information without relying solely on visual cues. By combining auditory messages with variable message signs’ content, drivers can receive timely alerts about potential hazards, even when their visual focus is elsewhere. What’s more, this integration would help drivers remain informed and ready to intervene when necessary, as auditory messages can act as a complementary means of alerting drivers. Ultimately, auditory messages can also serve as a backup communication channel in situations where visual displays may be obstructed or compromised, further reinforcing the reliability and effectiveness of the traffic system.

## Introduction

Human﻿ attention is essential to perform many daily activities properly, such as safely driving a vehicle. The concept of attention encompasses a number of functions and associated constructs, such as inattention, vigilance, or mind-wandering. Accordingly, different definitions, models, and taxonomies have been developed for these terms and will be briefly summarised.

*Driver inattention.* A specific taxonomy for inattention during driving has been proposed by Regan, Hallett & Gordon (2011). In accordance to these authors, the general concept of inattention occurs when there is insufficient attention or a total lack of attention towards critical activities for safe driving. Then, they postulate five different categories in which inattention may be displayed: driver restricted attention, driver misprioritised attention, driver neglected attention, driver cursory attention, and driver diverted attention. In the current study, we will focus on the particular case of the driver restricted attention (DRA). According to Regan et al. (2011), the DRA appears due to physical or biological factors, such as fatigue or micro-sleep, in situations where the driver has not rested well before driving, has been driving for a long time without rest, or must drive in monotonous environments (even if they had adequate attention conditions before driving).

*Vigilance.* We can define vigilance as the consistent ability to detect important stimuli over a long period of time (Hohmuth, 1970). Importantly, a typical phenomenon observed in tasks requiring vigilance is the decrease in vigilance performance as time goes by or when the task and/or environment are monotonous (Lemke, 1982; Pattyn et al., 2008). More generally, performance in vigilance tasks is subserved by the neurocognitive *alertness* network (Poster and Petersen, 1990; Poster, 2008), which is in control of both tonic alertness (i.e., sustained activation over a period of time) and phasic alertness (i.e., the increased response readiness for a short period of time subsequent to a *warning signal*). Interestingly, the use of warning signals could be a potential strategy to counteract vigilance decrement, as it will be further described in Section "Traffic messages as warning signals". In the specific context of driving, the inattention derived from a low vigilance state will be considered under the DRA category in Regan et al. (2011) taxonomy, as it is related to time-on-task and/or monotony, and the *vigilance decrement* has been frequently associated with traffic accidents (Pan et al., 2023).

*Mind-wandering.* Vigilance decrements are shown to be associated with mind-wandering, according to the subjective experience of participants (Baldwin et al., 2017; Pattyn et al., 2008). The phenomenon of mind-wandering is not new (Giambra, 1989), but it has attracted more attention in recent years (Baldwin et al., 2017; Berthié et al., 2015; Christoff & Fox, 2018). According to these studies, *mind-wandering* is an internal state of the individual where there is a shift of attentional focus from the task being performed to internal thoughts that are unrelated to the task at hand. Mind-wandering has been studied in the context of driving. For instance, Baldwin et al. (2017) studied this phenomenon during manual driving. Their participants drove a total of 40 min (20-min in a direction and other 20 in the reverse direction) along a monotonous freeway scenario. They found that the mind-wandering state had an important impact in the performance of drivers. In another example, Hidalgo-Muñoz et al. (2019) compared manual and autonomous driving and, as they expected, the mind-wandering state was more present during autonomous driving. The explanation of these authors was that autonomous driving represents a monotonous supervision task, as compared to manual driving (where the driver must be actively involved in the driving task).

Generally, human attention can be studied by means of behavioral data and/or psychophysiological measures. In particular, electroencephalography (EEG) is one of the most effective methods to assess the drivers’ attention states (Zhang & Eskandarian, 2020). As for the decrement of vigilance with time-on-task, studies have found that the power spectral density (PSD) of the alpha band, and also in the theta band, tend to increase with this decrement, whereas the density of the beta rhythm band tend to diminish with it (Guo et al., 2018; Larue et al., 2011; Mehrabi & Kim, 2022). However, the sensitivity of the beta band as a measure of the mental state in prolonged tasks has been questioned in meta-analyses (Tran et al., 2020). Interestingly for the current purposes, it has been proposed that the alpha power would be particularly sensitive to reflect low alertness due to monotony in a prolonged task (Wascher et al., 2016; Zhang & Eskandarian, 2020), and also that lower-frequency alpha (8–10.9 Hz) would be especially sensitive to reflect low alertness (Kamzanova et al., 2014; Klimesch, 1999).

Regarding mind-wandering, it has been also related to neurophysiological data (Baldwin et al., 2017; Hidalgo-Muñoz et al., 2019). Baldwin et al. (2017) found that, when driving manually, power in the alpha band of the EEG was higher in periods of self-reported mind-wandering, as compared to periods of self-reported on-task performance. Kam et al. (2022) developed a systematic review about the EEG signals of mind-wandering, and proposed that the most probable pattern of mind-wandering would be: (a) a greater activity of theta (4–8 Hz) and alpha (8–13 Hz) bands, while (b) a decrease in beta (13–30 Hz) band activity. However, to characterize mind-wandering and distinguish this particular state from other phenomena associated to low vigilance, like boredom (Pattyn et al., 2008), it is also important to take into account the participants’ self-reported answers about their perceived mind-wandering state (Hidalgo-Muñoz et al., 2019). Finally, in addition to the mind-wandering current state, it is also possible to assess the participant’s individual tendency to experience mind-wandering, for example by measuring the tendency to be attentive and aware of present-moment experience in daily life (Jermann et al., 2009).

### Partially automated driving and inattention

Driving on a highway with low cognitive stimulation seems to be a monotonous situation where the vigilance decrement could appear. Consequently, exploring potential countermeasures to reduce their occurrence is crucial, especially in the context of autonomous or partially autonomous driving.

One of the main reasons to develop autonomous or partially autonomous vehicles is to minimize human errors. According to the Society of Automotive Engineers (SAE), it is necessary to progress through six levels to achieve full automation of vehicles (SAE, 2021). Level 0 refers to the traditional manual cars, and level 5 represents fully automated cars of the future. From level 1 to level 5, the degree of automation increases progressively. For example, in level 1, the driver assistance system executes either the steering or the acceleration/deceleration functions for most of the time, while the human driver must supervise the system’s performance and handle the remaining aspects of the dynamic driving task. Currently, a considerable number of new vehicles sold would include a level 1 automation system. Importantly, being free from one or some of the driving subtasks may foster a vigilance decrement in the driver, which can affect the driver's attention to the road (Gold et al., 2013). One of the hypotheses to explain the vigilance decrement postulates that it is the consequence of the underarousal caused by the insufficient workload intrinsic to vigilance tasks (Dockree et al., 2007; Smallwood et al., 2004), which has been labelled as the “underload” view.

### Traffic messages as warning signals

A common way to increase drivers’ attention to relevant road and traffic events is by displaying such information on variable message signs (VMS). These signs can show messages about critical situations where the driver must, for example, regain manual control. Nevertheless, it could be argued that a driver experiencing the vigilance decrement may not properly attend to these messages, potentially resulting in critical circumstances not being detected with sufficient advance. In the current study, a particular strategy to counteract vigilance decrement is analyzed: the inclusion of in-vehicle auditory messages with the content of the VMS.

There is ample research about the use of auditory warning signals while driving in both manual (Ho & Spence, 2005; Singer et al., 2015; Wang et al., 2020), and autonomous driving (Baqapuri et al., 2023; Geitner et al., 2019; Kamizono et al., 2019; Kim et al., 2024; Lundkvist & Nykänen, 2016; Sawa et al., 2023; van der Heiden et al., 2018). One of the theoretical models that supports the applied research of driving warnings with different sensory modalities is the four-dimensional Multiple Resource Model (Wickens, 2008). According to this model, the simultaneous performance of two tasks is better if they rely on different sensory systems (e.g., visual vs. auditory) than on the same system. In addition to this theoretical advantage, the availability of the audio version of the VMS would present some others. First, audition is considered to be closely connected with brain’s arousal and activation systems (Meng & Spence, 2015). Second, the perception of auditory stimuli is “gaze-free” (Meng & Spence, 2015). Even though audition presents advantages, some problems may arise as well. For instance, these stimuli may be masked by background noise or music (Beruscha et al., 2011), or even, their processing be interfered with by secondary tasks that the driver might be engaged in (like using a mobile phone or talking to a passenger) (Kass et al., 2007; Mohebbi et al., 2009).

A few studies have examined the effects of receiving an additional auditory version of the VMS (Audio + Visual message modality) on the message processing, in manual driving (Tejero et al., 2020), or in distracted drivers of partially automated vehicles (Pi-Ruano et al., 2024). These studies have shown that drivers can discriminate between informative messages (i.e., general traffic knowledge or information about the traffic situation to come after some kilometers) and critical messages (i.e., important information about the current traffic situation) better in the Audio + Visual modality than in the absence of the auditory version of the message (i.e., in a Visual message modality). However, the potential effects of Audio + Visual VMS on the driver’s vigilance level was not examined in these previous studies.

### The current study

The rationale of the present study was that the use of Audio + Visual messages for relevant traffic information could: (a) help drivers to identify critical situations in which they must regain manual control, and (b) reduce the risk of experiencing low vigilance, even during periods of time beyond the presentation of the Audio + Visual messages. In other words, it could produce an indirect *alertness effect*, improving other driving-related subtasks. Therefore, we studied the utility of Audio + Visual messages and, particularly, we tested if those messages would serve as warning signals and, thus, they could have a lasting effect to reduce the vigilance decrement of the driver when the task demands are considerably low (partially autonomous driving in a monotonous environment). With that purpose, driving performance, electroencephalographic signals, and self-reported data were analyzed.

More specifically, the first goal of the present work was to add evidence that Audio + Visual messages would improve drivers’ performance to recognize the information on VMS, expanding the results reported by Tejero et al. (2020) and Pi-Ruano et al. (2024). In particular, the objective was to compare the participants’ accuracy and response distances in identifying Audio + Visual vs. Visual messages about critical situations in a monotonous driving situation. Secondly, we assessed if the availability of the audio versions would serve as warning signals and, thus, fostered a general higher level of attention (preventing the vigilance decrement), even in those specific moments where the audio was not being presented. This was tested by analyzing the different patterns of the theta, alpha and beta EEG bands of our participants while they were responding to a different driving subtask, such as reacting to the brake lights of a preceding vehicle. In addition, we analyzed if the mind-wandering phenomenon was associated to the vigilance decrement in the current study, by also considering self-reported data.

## Materials and methods

### Participants

The initial sample consisted of 43 adult volunteers (22 women), recruited through advertisements posted in social networks. All of them were native speakers of French; right-handed; with normal or corrected-to-normal vision; without previous history of reading difficulties, sensory impairments, or cognitive or neurological disorders; and with a driving licence for at least 3 years.

Four participants could not finish the experimental session: one of them due to some discomfort when using the driving simulator and the other three because of technical problems. Therefore, the final sample consisted of 39 participants (21 women) divided into two groups (see Section "Results" for further information).

The study was conducted according to the international ethical standards and was part of a larger research project positively evaluated by the Ethics Committee of Research in Humans at the University Gustave Eiffel (Univ. Eiffel) (October, 2021). In appreciation for participating in the study, each participant received a financial compensation of 50 €.

### Materials

#### Driving simulator

We used a Carnetsoft driving simulator (https://cs-driving-simulator.com/). The simulator was housed in a light- and sound-attenuated room of *The Ergonomics and Cognitive Sciences for Transport Laboratory* (LESCOT) of the Université Gustave Eiffel (Lyon, France). It included three 24-inch monitors, an operator interface monitor, and a set of driving controls consisting of a steering wheel, a steering gear box, and three pedals (Logitech G2). The central monitor was located at 38 cm from the participant’s eyes, so that the visual angle of the screen was 70° (on the horizontal axis) by 43° (on the vertical axis). The height of the seat was adjusted so that the line of sight of the participant fell on the focal point on the apparent horizon line in the driving environment displayed on this monitor. The other two simulation monitors were placed right next to the central one, one at each side. The simulator gave auditory feedback about the own vehicle engine and tires. The simulation software run on a personal computer (Intel Core i9-10850 3.6 GHz with a Nvidia RTX3080 10 GB graphic card). The participant’s actions on the vehicle controls, as well as speed and horizontal position of the participant’s vehicle on the road lane, were processed at a sample rate of 120 Hz, although continuous variables were downsampled by the simulator and recorded at 20 Hz.

#### Mind-wandering assessment

The tendency to experience mind-wandering was assessed at the beginning of the experiment with the French version of the Mindful Attention Awareness Scale (MAAS; Jermann et al., 2009). This scale contains 15 items to assess a core characteristic of dispositional mindfulness, namely, open or receptive awareness to what is taking place in the present (Brown & Ryan, 2003).

In addition, after each experimental block (see Section "Procedure"), the mind-wandering state was assessed with the self-reported answers of participants to the question about the proportion of thoughts related to the driving tasks or unrelated to them (by indicating a percentage – % – for each kind of thoughts).

### Tasks and stimuli

**Fig. 1:**
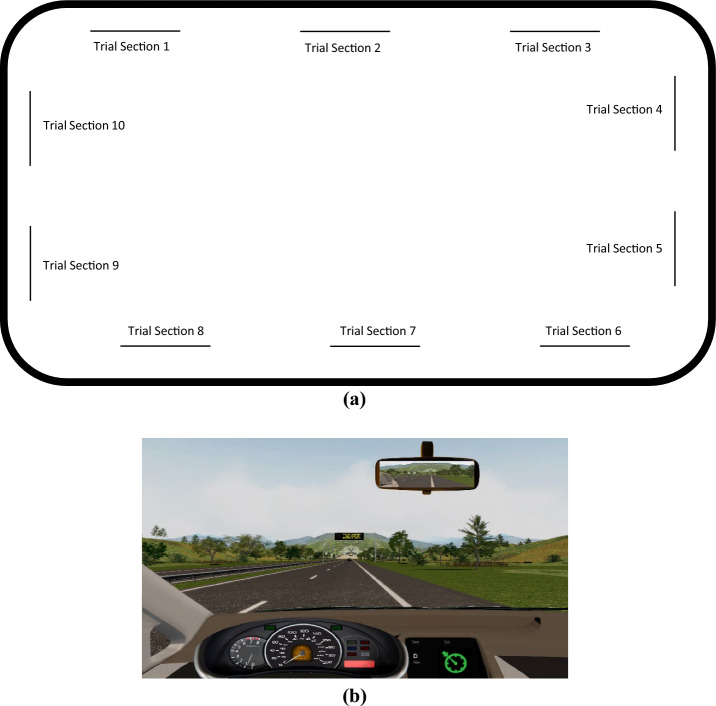
Circuit schema (**a**) and frontal view of the simulated scenery (**b**). **a** The line in bold represents the road, while the thin lines indicate the locations of the 10 trials. **b** The simulated scenery including an example of a variable message sign

Participants travelled along the right lane of a route in a simulated motorway environment (Fig. 1). We used a closed-loop track (see Fig. 1a) that mostly consisted of four straight road segments with two lanes on each direction and a speed limit of 110 km/h, which were connected by four curved sections with a speed limit of 80 km/h. Two of the four straight road segments were long enough to include three 350-m trial sections. The other two straight road segments were shorter and they encompassed two 350-m trial sections.

There were no other cars driving along the road (see Fig. 1b), with the exception of a preceding car (50 m in front of the participant’s) and a following car (50 m behind the participant’s). The former traveled at a constant speed along the route, except for the car-following trials (see Section "Tasks and stimuli"). The last one did not interact with the participant.

Participants were asked to drive along the simulated route while using the SAE level 1 automation mode. Therefore, for most of the time, the vehicle automatically controlled the speed while the participants manually controlled the steering wheel. In addition, participants were required to complete the VMS task and the car-following task described below, which were randomly presented through the route.

*VMS task*. The participants were asked to attend to the VMS along the road and recover full manual control of the car if the message presented on a VMS was a ‘critical message’, i.e., a message informing on circumstances that would require the driver to take over. In contrast, they had to keep on using the partially autonomous mode if the message presented was an ‘informative message’, i.e., a message informing on circumstances that would not require any action from the driver. Trials in the VMS task would show one of four ‘informative messages’ or the only one ‘critical message’ (see Table 1 for the original messages in French and their corresponding translations in English). In particular, the critical message was considered as the one that would require a lane change because of a sudden change in the road circumstances. On the contrary, the informative messages were considered as those that would not require a lane change.

**Table 1:** Text used in the VMS with an approximate translation

The four informative messages		The single critical message	
Original text	English translation	Original text	English translation
	Works on the right lane at 100 km		Works on the right lane at 100 m
	Lyon, stay on the right lane at 100 m		
	Works on the left lane at 100 m		
	End of works on the left lane 100 m away		

At the beginning of the trial, the message was not legible yet as there was a distance of 350 m to the VMS. In order to ensure the reading of the VMS, care was taken not to use messages that allowed completing the task by only reading a few words at the beginning or the end of the message. Specifically, as it is shown in Table 1, the critical message began with exactly the same words as in two of the informative messages (e.g. “Travaux sur voie de…”), and ended with exactly the same words as in two other informative messages (e.g. “… à 100 m”).

The participants were instructed to give the response at a distance to the VMS as far as possible, without making errors. The VMS were 3-D models of one of the most commonly used VMS on the French roads, allowing to display messages consisting of up to three lines of text, 15 or fewer characters each line. To represent the characters of text messages, we used the patterns available in CarDim (I.P.S. Vial, S.L.), a computer application aimed at creating sign models for traffic engineering projects.

In one of the message modality blocks of the experiment (see Section "Procedure"), we also used audio files to additionally present the text displayed on the VMS as an oral message (Audio + Visual message modality). Such audio files were prepared using a text-to-speech application available at http://fromtexttospeech.com/ (selecting the ‘French’ language, ‘Gabriel’ voice, and ‘medium’ speed), and were played through the audio system of the driving simulator. The audio started when the participant’s vehicle was just at a distance of 250 m to the VMS. According to pilot testing in the simulator, at such distance the text message on the VMS was not legible yet. As the audio message duration was about 2 s, the text message on the VMS was still not fully legible for the participant at the end of the audio message, which occurred when the participant’s vehicle was at a minimum distance of 189 m to the VMS. A concurrent presentation of the audio and visual messages was discarded to prevent possible interference effects that may lead the participants to focus on only one of them, depending on their preferences (Dalton et al., 2013; Liu, 2001).

The participants’ responses to take over vehicle control were given by pressing one of the two levers located behind the steering wheel, the most preferred one by each participant. After 2 s of full manual driving, they were fostered to recover the partially autonomous mode by pressing one of the two central buttons of the wheel. This indication was given by the flickering of a green pictogram in the dashboard and an auditory message. When the partially autonomous mode had been activated, the pictogram stopped flickering and remained steadily illuminated until the partially autonomous mode was deactivated again.

Trials in the VMS task ended when either: (a) the driver gave a response to the VMS, or (b) no response occurred and the driver reached the VMS location.

*Car-following task*. The preceding car would sometimes decelerate. The participants were asked to pay attention to this car to avoid crashes. In order to create a go/no-go task to assess vigilance, two different types of trials were designed.

On the one hand, short-brake light trials (SBLT) represented the neutral stimuli. During these trials, the preceding car did not actually decelerate but a short brake light appeared for 0.5 s. This was explained to participants as a fleeting pressing by the leading car’s driver as a result of an error, and so, it was not a dangerous situation. Consequently, no response was required in this sort of trials. On the other hand, long-brake lights trials (LBLT) represented the target trials. Here, the preceding car started to quickly decelerate with a deceleration rate of 2 m/s and the brake light appeared until the participants reacted. This was explained to participants as a dangerous situation where it was necessary to react quickly to avoid a crash. As a consequence, they were required to press the brake pedal to adjust their speed and maintain a constant distance to the preceding car. By pressing the brake pedal, the partially autonomous mode of the car was deactivated and our participants gained the full manual control of the vehicle. The ratio of appearance was 3 SBLT trials for each LBLT trial (see Section "Procedure" for a full description of trial type frequencies and ratios).

Trials in the car-following task ended when either: (a) the driver reacted to the brake light, or (b) no response was registered and the driver reached the spatial end of the trial (350 m after the beginning). The moment in which the leading car began to decelerate was randomized (between 197 and 13 m before the end of the trial) for each long-brake light trial, in order to avoid expectancy effects. As in the VMS task, the participants were fostered to recover the partially autonomous mode after some seconds of manual driving.

Finally, catch-trials (i.e., trials with no VMS and no brake event) were also included so as to generate uncertainty on whether the previous car would actually brake.

### Procedure

Firstly, the participants read and signed an informed consent form. Then, they filled in a questionnaire asking for some sociodemographic data and for information on driving habits, as well as the MAAS questionnaire. Next, the participants got comfortable in the driver’s seat and the experimenter put on them the corresponding cap and the electrodes for EEG recording.

Then, they completed a training on the VMS task, with no simultaneous driving, on which the critical and informative messages were presented on the central monitor, one at a time, at least four times each, in different orders: (1) Visual messages, sequential presentation (critical and informative messages not mixed), (2) Audio + Visual messages, sequential presentation, (3) Visual messages, random presentation (critical and informative message mixed), (4) Audio + Visual messages, random presentation.

After that, each participant completed the route twice, separated by a 10-min break to rest. During one block, the messages were only available as text messages on the VMS (Visual message modality). During the other block, oral message versions of the text VMS were additionally presented (Audio + Visual message modality), as described in Section "Procedure". The order of these two conditions was counterbalanced between both groups. In each message modality block, three initial training trials with blank VMS were incorporated for participants getting used to the simulator, which were followed by ten practice trials: five to practice the VMS task, four to practice the car-following task, and one catch-trial. After these practice trials, 99 experimental trials were randomly presented for each participant in each message modality block: 50 VMS task trials (5 messages × 10 repetitions), 40 car-following task trials (10 repetitions for the long-brake light trials and 30 repetitions for the short-brake light trials), and 9 catch-trials. Consequently, participants completed 112 trials per message modality block. On average, participants took about 90 min to complete the two drives. Driving was only stopped during the 10-min break between the two experimental blocks. Finally, after each block the participants answered the question about the mind-wandering state. Twenty participants went through the Visual block first (Visual First group) and 19 participants went through the Audio + visual block first (Audio + Visual First group).

### Measures, design, and statistical analyses

#### Driving performance

Data from trial sections were analyzed. The experimental design included two manipulated variables: message modality as a within-subject factor (Visual vs. Audio + Visual) and the counterbalance order as a between-subject factor (Visual First vs. Audio + Visual First).

For the VMS task, we obtained the following measures for each participant:

- M1: the Signal Detection Theory (SDT) measure of sensitivity to discriminate critical and non-critical messages, *d’*.
- M2: the SDT measure of response bias, to know the willingness of participants to indicate that the critical message was present, *β*.
- M3: the average response distance, defined as the mean distance from the participant’s car to the VMS when the response in the VMS task was given (in meters). This measure was computed excluding the trials on which the participant gave a wrong response.

For the car-following task, we obtained the subsequent measures for each participant:

- M4: sensitivity to discriminate long and short brake lights, *d’*.
- M5: response bias, the willingness to indicate the presence of long brake lights, *β*.
- M6: average response latency, defined as the time from the appearance of the brake light to the participants’ response (in seconds). Wrong response trials were excluded.

All these measures were averaged per participant and message modality, and analyzed by separate analyses of variance (ANOVA), which were performed using the IBM SPSS Statistics 26 software.

#### Electroencephalographic data

The electrophysiological data were recorded with the BioSemi ActiveTwo System (http://www.biosemi.com/). For both EEG and EOG, signals were acquired using an ActiveTwo Ad box, 24-bit ADC per channel sampled at 1024 Hz.

An electrode cap containing 34 active electrodes with placement based on the International 10–20 System (Fz, Cz, Pz, Oz; Fp1, F7, F3, FC1, FC5, T7, C3, CP1, CP5, P7, P3, PO3, O1, IMa; midway Oz-Ma1) and their counterparts on the right hemi-scalp, Ma1 and Ma2 (left and right mastoids, respectively) was used. Electrooculography activity (EOG) was recorded from the outer canthus of the right eye so as to filter epochs occurring during eye movements which could prevent sufficient sensory information of the signal.

The power spectral density (M7) during the five previous seconds to the appearance of brake-lights was analyzed. As short and long brake lights were undistinguishable at the instant they were presented, both types of brake lights were indistinctly considered for this analysis.

We used Matlab, EEGLAB, and Linear Modeling (LIMO; Maris & Oostenveld, 2007; Pernet et al., 2015) to import, filter, remove Independent Component Analysis (ICA) subcomponents, epoch and build our design. A two-pass band-pass filter (1 – 40 Hz) was applied. Epochs were created by selecting 5-s intervals before each brake light (80 per participant). ICA blinks subcomponents were discarded after visual inspection. Heavily contaminated epochs by muscle artifacts were also discarded after visual inspection (on average, 6 epochs discarded per participant).

#### Self-reported mind-wandering state

We analyzed the answers that the participants gave to the questions about their perceived mind-wandering state (M8) during each driving block: percentage of unrelated thoughts to the tasks.

## Results

Table 2 shows descriptive statistics and *t* tests of sociodemographic data and assessment variables for the two groups. They did not significantly differ in age or driving experience, while they did differ in their prior tendency to mind-wander.

**Table 2:** Descriptive statistics (M = mean, SD = standard deviation) of sociodemographic data and assessment variables for the Visual First and Audio + Visual First groups, and results from Student’s *t* tests

Measure	Counterbalance group				*t* tests		
	Visual first		Audio + visual first		*t*_(1,37)_	*p*	Cohen’s *d*
	M	SD	M	SD			
Age	27.7	4.7	26.6	4.1	0.79	0.433	0.254
Driving experience (years)	8.4	4.6	7.6	4.2	0.58	0.562	0.187
Mind-wandering tendency	40.7	12	48.8	10	− 2.31	0.027	11.09

### Behavioral results

#### VMS task

Table 3 shows some descriptive statistics of the behavioral measures obtained.

**Table 3:** Percentage of correct responses and mean values across participants of the other measures obtained during the experimental trials (standard deviation between parentheses, 95% CI between brackets), by group (Visual First/Audio + Visual First) and message modality (Visual/Audio + Visual)

	Visual first	Audio + visual first		
	Visual	Audio + visual	Visual	Audio + visual
Correct responses (%)	97.40 (0.16) [96.21, 98.29]	99.80 (0.05) [99.28, 99.98]	98.21 (0.13) [97.15, 98.95]	99.16 (0.09) [98.35, 99.64]
False alarms (%)	0.032 (0.019) [0.024, 0.040]	0.013 (0.003) [0.011, 0.015]	0.022 (0.016) [0.013, 0.030]	0.016 (0.006) [0.014, 0.019]
*d*′	3.48 (0.43) [3.31, 3.66]	3.84 (0.10) [3.78, 3.92]	3.60 (0.33) [3.42, 3.78]	3.75 (0.18) [3.68, 3.82]
*β*	2.12 (0.94) [1.38, 2.86]	3.28 (0.63) [2.93, 3.64]	3.17 (2.12) [2.42, 3.93]	2.97 (0.91) [2.61, 3.34]
Average response distance (m)	69.9 (19.4) [60.3, 79.6]	156.1 (13.9) [150.4, 161.8]	64.8 (23.2) [54.9, 74.7]	160.0 (10.7) [154.2, 165.8]

*M1. Sensitivity (d′)*. Only the main effect of message modality was significant (*F*(1, 37) = 16.64, *p* < 0.001, η^2^_p_ = 0.310). The ability to discriminate critical and non-critical VMS messages was higher in the Audio + Visual modality (M = 3.80, SD = 0.14) than in the visual one (M = 3.54, SD = 0.38). The interaction and the main effect of group were not significant (interaction: *F*(1, 37) = 2.98, *p* = 0.093, *η*^2^_p_ = 0.074; and group: *F*(1, 37) = 0.030, *p* = 0.863, *η*^2^_p_ = 0.001).

*M2. Response bias (β).* The interaction between the message modality and the group was significant (*F*(1, 37) = 5.31, *p* = 0.027, *η*^2^_p_ = 0.126). Participants in the Audio + Visual First group did not change their response criteria in neither of the message modalities (mean difference = 0.20). However, participants in the Visual First group did change their criteria when facing the Audio + Visual modality (mean difference = -1.17). Main effects were not significant (message modality: *F*(1, 37) = 2.660, *p* = 0.111, *η*^2^_p_ = 0.067; and group: *F*(1, 37) = 1.762, *p* = 0.193, *η*^2^_p_ = 0.045).

**Fig. 2:**
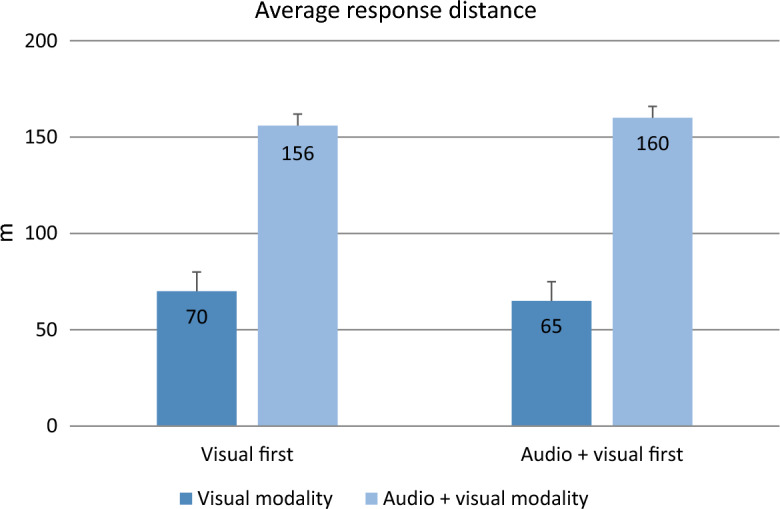
Average distance of responses (m) for each group (Visual First vs. Audio + Visual First) and message modality (Visual vs. Audio + Visual). Error bars indicate the standard error (SE)

*M3. Average response distance*. Only the main effect of message modality was significant (*F*(1, 37) = 1056.932, *p* < 0.001, *η*^2^_p_ = 0.966). The participants responded correctly more quickly in the Audio + Visual modality (M = 158 m, SD = 12.3) than in the visual one (M = 67 m, SD = 21.3) (see Fig. 2). The interaction and the main effect of group were not significant (interaction: *F*(1, 37) = 2.639, *p* = 0.113, *η*^2^_p_ = 0.067; and group: *F*(1, 37) = 0.017, *p* = 0.896, *η*^2^_p_ < 0.001).

#### *Car*-following task

Table 4 shows some descriptive statistics of the measures obtained.

**Table 4:** Percentage of correct responses and mean values across participants of the other measures obtained during the experimental trials (standard deviation between parentheses, 95% CI between brackets), by group (Visual First/Audio + Visual First) and message modality (Visual/Audio + Visual)

	Visual first	Audio + visual first		
	Visual	Audio + visual	Visual	Audio + visual
Correct responses (%)	100 (0.0) [100, 100]	99.50 (2.24) [98.77, 100]	100 (0.0) [100, 100]	100 (0.0) [99.25, 100]
False alarms (%)	0.018 (0.004) [0.010, 0.025]	0.018 (0.004) [0.000, 0.049]	0.027 (0.024) [0.019, 0.035]	0.053 (0.099) [0.021, 0.086]
*d*′	3.76 (0.07) [3.66, 3.85]	3.73 (0.15) [3.56, 3.91]	3.63 (0.28) [3.54, 3.73]	3.54 (0.54) [3.36, 3.72]
*β*	2.44 (0.25) [2.21, 2.68]	2.51 (0.09) [2.26, 2.77]	2.19 (0.68) [1.96, 2.43]	2.14 (0.80) [1.88, 2.40]
Average response latency (s)	2.2 (0.50) [1.972, 2.427]	2.1 (0.44) [1.909, 2.364]	1.8 (0.49) [1.596, 2.063]	1.8 (0.56) [1.552, 2.020]

*M4. Sensitivity (d′)*. Neither the interaction (*F*(1, 37) = 0.560, *p* = 0.459, *η*^2^_p_ = 0.015) nor the main effect of message modality (*F*(1, 37) = 1.575, *p* = 0.217, *η*^2^_p_ = 0.041), nor the main effect of group (*F*(1, 37) = 3.10, *p* = 0.086, *η*^2^_p_ = 0.077) were significant.

*M5. Response bias (β).* Neither the interaction (*F*(1, 37) = 1.92, *p* = 0.174, *η*^2^_p_ = 0.049) nor the main effect of message modality (*F*(1, 37) = 0.039, *p* = 0.845, *η*^2^_p_ = 0.001), nor the main effect of group (*F*(1, 37) = 3.53, *p* = 0.068, *η*^2^_p_ = 0.087) were significant.

**Fig. 3:**
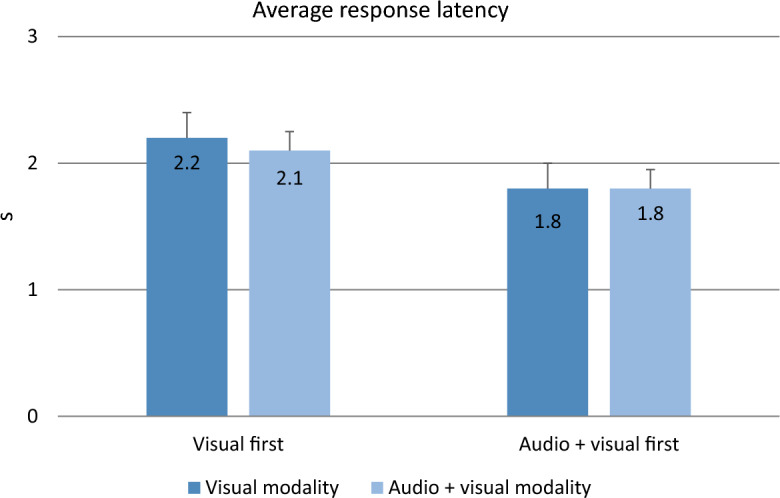
Average response latency (s) for each group (Visual First vs. Audio + Visual First) and message modality (Visual vs. Audio + Visual). Error bars indicate the standard error (SE)

*M6. Average response latency*. Only the main effect of group was significant (*F*(1, 37) = 5.525, *p* = 0.024, *η*^2^_p_ = 0.130). Participants in the Audio + Visual First group responded correctly more quickly (M = 1.8 s, SD = 0.53) than participants in the Visual First group (M = 2.15 s, SD = 0.47) (see Fig. 3). The interaction and the main effect of message modality were not significant (interaction: *F*(1, 37) = 0.040, *p* = 0.843, *η*^2^_p_ = 0.001; and message modality: *F*(1, 37) = 1.154, *p* = 0.290, *η*^2^_p_ = 0.030).

### EEG spectra

These analyses were performed for 31 participants (16 women), as 8 people were excluded due to a noisy signal registration: 16 (8 men and 8 women) in the Visual First group and 15 (7 men and 8 women) in the Audio + Visual First group.

**Fig. 4:**
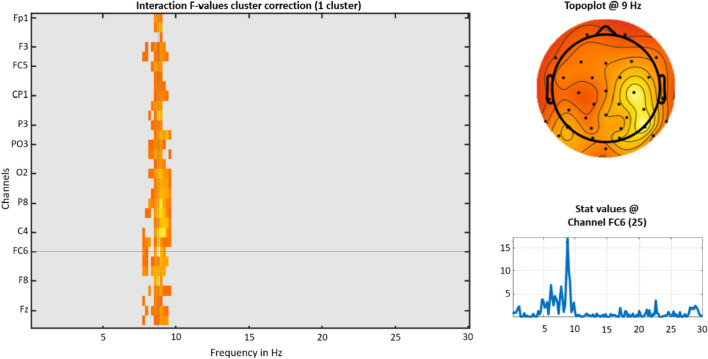
EEG power spectrum in LIMO for the whole scalp, preceding the appearance of brake lights. The graph on the left shows the resulting frequency range of the interaction cluster. The plots on the right show the exact frequency and channel of the maximum value of the cluster

Power in the theta, alpha, and beta frequencies throughout the scalp were selected for analysis. No effects reached significance for theta and beta power. However, an interaction was found for alpha power between group and message modality, *p* = 0.024 (see Fig. 4). The cluster found represents the width of the frequency bin (starting at 7.8 Hz and ending at 9.6 Hz) defined as alpha.

**Fig. 5:**
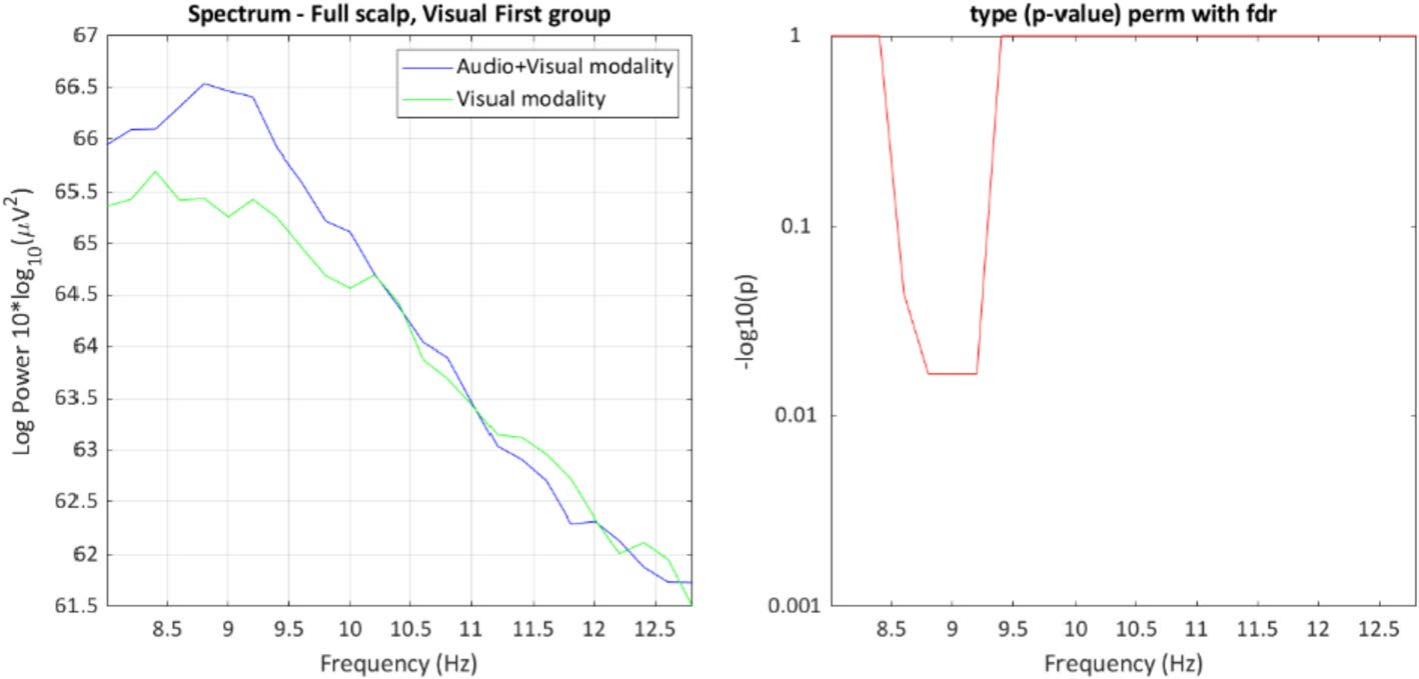
Simple effect of the alpha band power in the Visual First group. The graph on the left shows the descriptive values of the power spectrum. The graph on the right displays the *p* value between both messages modalities

**Fig. 6:**
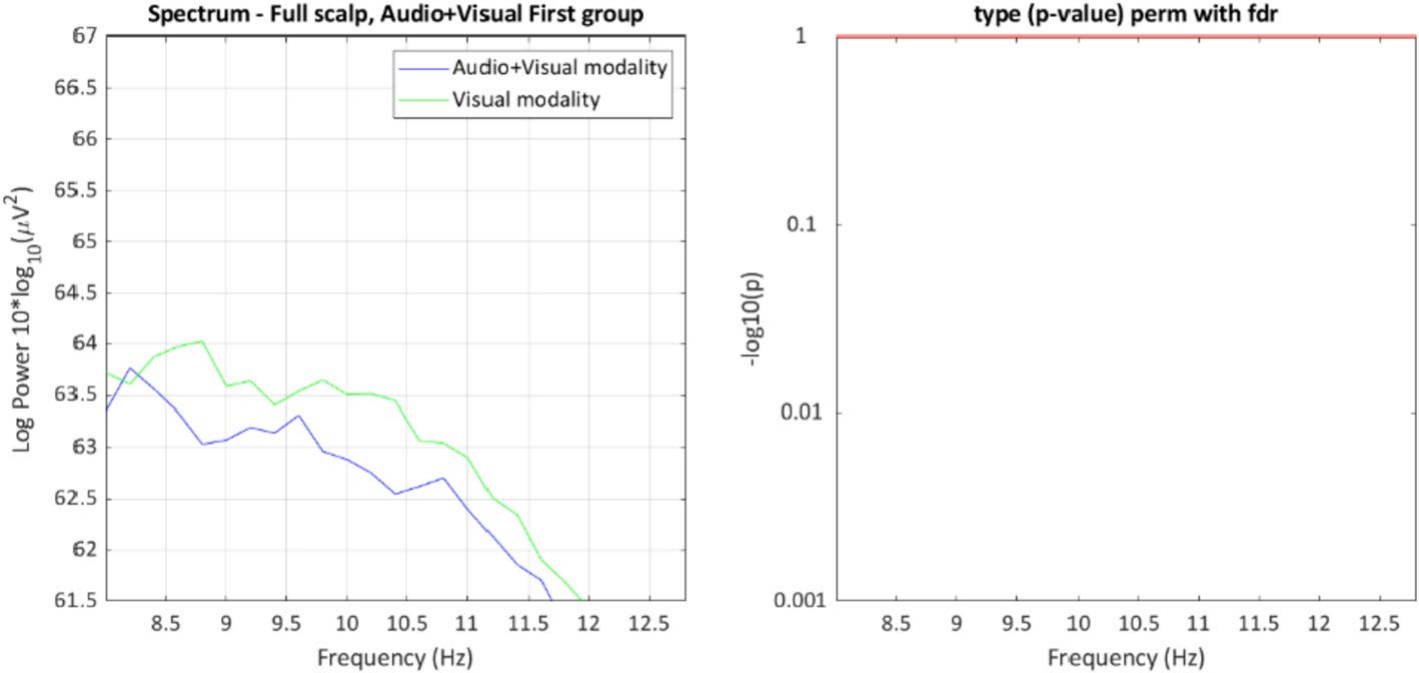
Simple effect of the alpha band power in the Audio + Visual First group. The graph on the left shows the descriptive values of the power spectrum. The graph on the right displays the *p* value between both messages modalities

To further analyze the reported interaction, Figs. 5 and 6 show the simple effects of message modality across the levels of group. Participants in the Visual First group (Fig. 5) showed less alpha power during the Visual message modality than in the Audio + Visual one. Participants in the Audio + Visual First group (Fig. 6) showed no statistical differences between both message modalities.

### Self-reported mind-wandering state

No significant differences were found for the self-reported percentages of mind-wandering, as measured at the end of each experimental block: (a) neither for the interaction (*F*(1, 37) = 0.142, *p* = 0.708, *η*^2^_p_ = 0.004), (b) nor for the main effect of other thoughts percentages (*F*(1, 37) = 2.248, *p* = 0.142, *η*^2^_p_ = 0.057), (c) nor for the main effect of group (*F*(1, 37) = 0.253, *p* = 0.618, *η*^2^_p_ = 0.007).

## Discussion

The first aim of this study was to provide further evidence regarding the potential benefits of presenting audio messages to complement the information provided by text messages on VMS. In particular, our results would be useful to discuss if the previously reported advantage of the Audio + Visual messages could be also observed, particularly, when people find themselves in a monotonous driving situation. Then, the second objective was to assess whether the availability of the audio versions could serve as warning signals and, thus, produce an indirect *alertness effect*, improving drivers’ performance in other driving subtasks not related to the message processing (i.e., a car-following task). In summary, the observed results would provide positive evidence to support the use of audio messages to complement text on VMS, which would help drivers to (a) better process the traffic information shown on VMS and (b) keep a higher level of alertness along the driving time.

First, in the VMS task, the drivers responded earlier and more accurately (higher sensitivity — d′) in the Audio + Visual message modality. That is to say, participants were able to discriminate the critical and non-critical VMS messages more correctly and at longer (i.e., safer) distances in the Audio + Visual message modality as compared to the Visual one. These results are consistent with what was found in Tejero et al. (2020) and Pi-Ruano et al. (2024), expanding this evidence to a different driving situation (i.e., monotonous driving), a different population of drivers (i.e., French drivers), and an experimental setting with other driving demands (i.e., incorporating a car-following task), other VMS messages, and a different simulated route. In addition, the present study contributes to the previous literature supporting that auditory signals could be advantageous in different contexts, such as automated driving (Geitner et al., 2019), distraction (Han & Ju, 2021; Parmentier et al., 2008; SanMiguel et al., 2008), alertness and preparation (Petersen & Posner, 2012), and drowsiness (Han & Ju, 2021).

In regard to the response bias (*β*), participants in the Visual First group completed the first block (Visual) with a more liberal criterion, becoming more conservative in the second block (Audio + Visual). In contrast, participants in the Audio + Visual First group kept a similar criterion in the first (Audio + Visual) and second (Visual) blocks. Previous evidence (Luna et al., 2018; Thomson et al., 2016) has shown that the vigilance decrement is often associated to an increment in response bias, rather than a loss in sensitivity. Therefore, the change towards a more conservative criterion in the Visual First group would reflect the expected decrease in vigilance associated to a monotonous driving task (Borghini et al., 2014; Parasuraman, 1986; Warm et al., 2008), such as the one used in the current experiment. In contrast, no significant change in the response bias was observed in the Audio + Visual First group, suggesting that the vigilance decrement expected in the second block could had been counteracted in this particular group condition. This result is consistent with the idea of an increased alertness effect (Petersen & Posner, 2012) induced by the Audio + Visual stimuli that would partially persist along the second block, mitigating the expected vigilance decrement observed in the VMS task. Research has shown that auditory stimulation, among other strategies, help reducing the decrement in vigilance (see Al-Shargie et al., 2019 for a review). In fact, these authors proposed a new auditory stimulation method (exposure to a pure tone at 250 Hz) and found that it was effective in improving the vigilance level (Al-Shargie et al., 2021). Nevertheless, tones can only alert, while audio messages would have the advantage of both alerting and making VMS information more accessible.

Regarding the car-following task, participants reacted faster to the brake lights when they performed the Audio + Visual message modality first and then kept a similar reaction time even when they next completed the Visual message modality. In contrast, participants in the Visual First group reacted slower to the brake lights in both the Visual and the Audio + Visual blocks, as compared to the other group. These results provide partial evidence of our hypothesis, as we expected an interaction between group and message modality. The fact that the quicker average reaction time to the brake lights remains unaffected in the Visual block when performed after the Audio + Visual block is consistent with the idea of an increased alertness effect induced by the Audio + Visual stimuli that would partially persist along the second block, mitigating the expected vigilance decrement whose impact should have been visible in the car-following task. But we also expected a more patent change in performance in the Audio + Visual block when performed after the Visual block. To explain the latter result, we must consider that performance in the second experimental block would be influenced by two opposite factors. First, performance would decrease after completing a long and monotonous task, which would be reflected by the typical pattern of slower reaction times that take place during the vigilance decrement (Esterman et al., 2014; Greenlee et al., 2018; Gyles et al., 2023; Hilti et al., 2013; Luna et al., 2022; Satterfield et al., 2019). Second, Audio + Visual messages would make the task slightly more stimulating (Dietrich & Prior, 2016; Enders et al., 2006; Parmentier et al., 2008; SanMiguel et al., 2008), increasing performance with faster reaction times. As consequence, these two opposite factors would cancel each other in the Audio + Visual message modality of the Visual First group (when it is completed in the second block), but this cancelation would not occur in the Audio + Visual First group.

The power spectral analysis provided further evidence to support the proposed increased alertness effect. As previously described, an interaction between group and message modality was found for the alpha band, with a significant cluster starting at 7.8 Hz and ending at 9.6 Hz, i.e., around what some researchers have called low alpha (Klimesch, 1999). This interaction revealed that participants in the Visual First group showed an increased alpha power in the second block (Audio + Visual) as compared to the first block (Visual), which is consistent with the expected decrease in vigilance (Borghini et al., 2014; Kamzanova et al., 2014; Sauseng et al., 2006; Simon et al., 2011). In contrast, participants in the Audio + Visual First group kept a similar alpha power in the first (Audio + Visual) and second (Visual) blocks. Again, the fact that the alpha power was not increased in the Visual block when performed after the Audio + Visual block is consistent with the idea of an increased alertness effect induced by the Audio + Visual stimuli that would partially persist along the second block, mitigating the expected vigilance decrement in the car-following task (Antons et al., 2013; Kuribayashi & Nittono, 2017; Sun et al., 2016).

Finally, regarding mind-wandering, the power spectral analysis showed no differences for the theta and beta bands, only for the alpha band. These results do not fully reflect the neuropsychological pattern for mind-wandering proposed by Kam et al. (2022), and thus, our data would not support that our participants were particularly experiencing mind-wandering. In fact, the subjective, self-reported measure of the mind-wandering state was found to be similar across groups and message modalities. This means that participants would not find any difference in their subjective feeling of mind-wandering, although they were actually experiencing low vigilance, as suggested by the behavioural and neuropsychological data previously discussed.

### Limitations

The current study has some relevant limitations that could impact the interpretation of the results. First, no differences in the mind-wandering state were found between message modalities. However, the nature of our messages and tasks could have influenced the absence of such state. Maybe, if different tasks had been chosen, or a neutral condition (no message) had been included, it would have been easier to find mind-wandering. In addition, it should be noted that the groups of participants did actually differ in their prior tendency to mind-wander, as evidenced by the MAAS scale. Still, no evidence of differences in mind-wandering were found after each experimental block, as previously discussed. Therefore, despite any difference in the prior tendency to mind-wander, our data are not consistent with the idea of the mind-wandering state having a significant influence in the reported results.

Second, comparing the separate effects of verbal and non-verbal stimuli was not a specific objective of the current study. Therefore, we cannot rule out, with the evidence provided, that other non-verbal stimuli would potentially generate similar effects as the ones reported in the current study (e.g., alerting effects could also be expected by using different kinds of warning signals, such as pure tones, visual prompts or haptic cues, as well as with verbal auditory messages). For instance, in future studies, drivers could be trained to react to certain auditory signals that will cue motorists to pay attention when critical information is being relayed to them. However, we think that there are potential advantages of using, specifically, verbal auditory messages. For example, in-vehicle verbal auditory messages can anticipate relevant traffic information, as they can be presented before the text and pictograms on a VMS are actually visible or, even, when they are not fully visible (e.g., due to adverse weather conditions or VMS malfunctioning). In fact, in-vehicle verbal messages would allow reconsidering the way traffic administrations provide dynamic information to drivers, as it is technologically feasible to present such messages at any specific location of the road (not necessarily where a VMS is placed). Perhaps some of such messages could be successfully coded as text and/or pictograms presented in-vehicle, but more complex or unstandardized messages would be better transmitted auditorily, considering that a complex or unexpected text message would require diverging the eyes from the road during a sustained reading time.

In addition, with the experimental design of the current study, it is not possible to determine the duration of the proposed mitigation effect of the Audio + Visual messages on vigilance. Future studies should explore the duration and benefits of each strategy more thoroughly.

We can also add that only one experiment was reported. Therefore, further research will be useful to confirm the effects reported using different groups of drivers and to verify that no other relevant effects would also be found with larger samples. Finally, our data were gathered using a driving simulator, and thus, real-driving studies would be required to expand our results to real-life settings.

## Conclusion

According to our results, audio + visual messages would help drivers to (a) better process traffic information shown on VMS compared to the visual messages, and (b) keep a higher level of alertness along the driving time, in particular, in a situation of partially autonomous driving. But, can we actually provide the content of a VMS as a complementary in-vehicle auditory message? As far as current technologies are empowering connected vehicles, such complementary messages are indeed a reality (see, for example, READit VMS application; https://links.uv.es/lectura/readitvms).

## Data Availability

The datasets generated and/or analysed during the current study are available in the Open Science Framework (OSF) repository, https://osf.io/rtq4w/.
